# Managing the risk of lithium-induced nephropathy in the long-term treatment of patients with recurrent affective disorders

**DOI:** 10.1186/1741-7015-11-34

**Published:** 2013-02-11

**Authors:** Emanuel Severus, Michael Bauer

**Affiliations:** 1 Department of Psychiatry and Psychotherapy, University Hospital Carl Gustav Carus, Technische Universität Dresden, Fetscherstr. 74, D-01307 Dresden, Germany

**Keywords:** lithium, affective disorders, chronic kidney disease, shared decision making.

## Abstract

Lithium has been the most effective psychopharmacological drug in the long-term treatment of patients with recurrent unipolar and bipolar affective illness. As a result of its widespread and longtime use in patients with recurrent affective disorders, psychiatrists have become increasingly aware of the whole spectrum of lithium's potential side effects. One of the side effects associated with its chronic use is lithium-induced nephropathy. In a recent cross-sectional study published in *BMC Medicine*, Alberto Bocchetta *et al*. add further information to this topic, demonstrating that duration of lithium treatment is associated with impaired glomerular function in patients with recurrent or chronic affective disorders. The present paper will discuss the implications of this and other related recent research on our management of patients with recurrent affective disorders. In this context the importance of shared decision making and close monitoring of kidney function is highlighted, including the regular assessment of the glomerular filtration rate, to provide best possible care to our patients maintained on lithium treatment.

See related research article here http://www.biomedcentral.com/1741-7015/11/33

## Background

For the last few decades lithium has been the most effective psychopharmacological drug in the long-term treatment of patients with recurrent unipolar and bipolar affective illness [1]. As a result of its widespread and longtime use in individual patients with recurrent affective disorders, psychiatrists have become increasingly aware of the whole spectrum of lithium's potential side effects. One of the side effects associated with its chronic use is lithium-induced nephropathy (with tubulointerstitial nephritis being the characteristic histopathological finding), which may finally manifest itself as end-stage renal disease, in need of either dialysis or kidney transplantation [2].

In a recent cross-sectional study published in *BMC Medicine*, Alberto Bocchetta *et al*. add further information on this topic, demonstrating that the duration of lithium treatment is associated with impaired glomerular function in patients with recurrent or chronic affective disorders [3]. In their study, for each year of lithium treatment the estimated glomerular filtration rate (eGFR) was estimated to decrease by 0.64 mL/minute on average, in addition to the adverse effects of advancing age. In patients who had been on lithium for at least 12 months, chronic kidney disease (defined by an eGFR below 60 mL/minute) was present in about 1 out of 4 patients, compared to 1 out of 18 in lithium-naïve patients. While age differences may have contributed to the latter finding (the lithium treated patients were on average eight years older than the lithium-naïve patients) these results are in line with similar data from different research groups during previous years [4,5]. Finally, in a recent meta-analysis lithium treatment was associated with a reduction in GFR ranging from 0 to 5 mL/minute over a mean observation time of one year [6]. While 5 mL/minute represent only 5% of the minimum normal GFR, this may well become clinically relevant in patients on long-term lithium treatment within less than a decade as an eGFR below 60 mL/minute is already associated with an increased risk for all-cause mortality and end stage renal disease [7,8].

## Close monitoring of patients on lithium therapy

What are the implications of these findings for the long-term treatment of patients being treated with lithium in clinical practice? First of all, kidney function should be carefully assessed in every patient for whom lithium is considered a viable treatment option and before treatment is started. In an inpatient setting the measurement of creatinine clearance is recommended as it usually accurately reflects glomerular filtration rate and complete 24-hour urine collection is feasible. In an outpatient setting this may be more difficult; therefore, measuring serum creatinine to calculate the eGFR may represent a reasonable alternative although it is considered less precise in the early stages of chronic kidney disease [9]. The eGFR is either provided directly by the laboratory or can be calculated with the help of internet-available calculators, for example, from the UK CKD eGuide on the Renal Association website [10,11]. Compared to the measurement of serum creatinine alone both parameters are more sensitive to detecting mild renal insufficiency [4]. If a patient shows values within the normal range in these assessments and is put on lithium thereafter, serum creatinine to calculate eGFR should be assessed at least twice a year, and more frequently if indicated, supplemented by urinanalysis to assess urine concentration ability and proteinuria. If eGFR falls below 60 mL/minute/1.73 m^2 ^(generally referred to as chronic kidney disease) a nephrologist should be consulted for discussion and more comprehensive evaluation. Whether to stop lithium in a patient with chronic kidney disease should be done in a shared decision making process, including a discussion of alternative treatment options with the patient. Importantly, stopping lithium may only have beneficial effects for the kidney as long as the creatinine clearance is > 40 mL/minute, otherwise the underlying pathology may progress despite the elimination of the triggering toxic compound (lithium), with parallel deterioration of kidney function at the same rate as before [12].

## Conclusions

In conclusion, while lithium remains one of the most effective treatment options in the long-term treatment of patients with recurrent affective disorders, monitoring of kidney function at least twice a year is mandatory and should include the assessment of the glomerular filtration rate either by 24-hour creatinine clearance or eGFR.

## Abbreviations

eGFR: estimated glomerular filtration rate.

## Competing interests

In the past five years ES has been on the speakership bureaus of AstraZeneca, Bristol-Myers Squibb, Eli Lilly and Company and Lundbeck. In addition he is a member of the International Group for the Study of Lithium Treated Patients (IGSLI). In the past five years MB has received grant/research support from The Stanley Medical Research Institute, Deutsche Forschungsgemeinschaft and the European Commission (FP7). He is a consultant for AstraZeneca, Eli Lilly, Servier, Lundbeck, BMS and Otsuka, Takeda. He has received speaker honoraria from AstraZeneca, Lilly Deutschland, Lundbeck, Servier, Pfizer, BMS and Otsuka.

## Authors' contributions

ES and MB contributed to the conceptualization, drafting and editing of this manuscript. Both authors have read and approved the final manuscript.

## Authors' Information

ES is head of the affective disorders inpatient unit at the Department of Psychiatry and Psychotherapy at the University Hospital Carl Gustav Carus, Technische Universität Dresden. He is member of the International Group for the Study of Lithium Treated Patients (IGSLI) and Managing Editor of *International Journal of Bipolar Disorders*. MB is Professor of Psychiatry, Director and Chairman of the Department of Psychiatry and Psychotherapy at the University Hospital Carl Gustav Carus, Technische Universität Dresden. He is the President of the International Group for the Study of Lithium Treated Patients (IGSLI) and Editor - in - Chief of *International Journal of Bipolar Disorders*.

## Pre-publication history

The pre-publication history for this paper can be accessed here:

http://www.biomedcentral.com/1741-7015/11/34/prepub
